# Traditional Tibetan medicine to fight against COVID-19: Basic theory and therapeutic drugs

**DOI:** 10.3389/fphar.2023.1098253

**Published:** 2023-02-16

**Authors:** Kun Zhang, Lijie Wang, Jiayan Peng, Kangzhuo Sangji, Yuting Luo, Yujiao Zeng, Yongzhong Zeweng, Gang Fan

**Affiliations:** ^1^ State Key Laboratory of Southwestern Chinese Medicine Resources, School of Ethnic Medicine, Chengdu University of Traditional Chinese Medicine, Chengdu, China; ^2^ School of Pharmacy, Chengdu University of Traditional Chinese Medicine, Chengdu, China

**Keywords:** traditional Tibetan medicine, herbal medicine, COVID-19, basic theory, therapeutic drug

## Abstract

The Coronavirus Diseases 2019 (COVID-19) has been rapidly spreading globally and has caused severe harm to the health of people and a substantial social burden. In response to this situation, experts around the world have considered various treatments, including the use of traditional medicine. Traditional Tibetan medicine (TTM), one of the traditional medicines in China, has played an important role in the treatment of infectious diseases in history. It has formed a solid theoretical foundation and accumulated rich experience in the treatment of infectious diseases. In this review, we provide a comprehensive introduction to the basic theory, treatment strategies, and commonly used drugs of TTM for the treatment of COVID-19. In addition, the efficacies and potential mechanisms of these TTM drugs against COVID-19 are discussed based on available experimental data. This review may provide important information for the basic research, clinical application and drug development of traditional medicines for the treatment of COVID-19 or other infectious diseases. More pharmacological studies are needed to reveal the therapeutic mechanisms and active ingredients of TTM drugs in the treatment of COVID-19.

## 1 Introduction

In the late December 2019, an outbreak of unexplained pneumonia began in Wuhan, Hubei province, China. The epidemic disease rapidly spread worldwide and posed a significant threat to the lives of billions of people (Lu et al., 2020). Soon after, the world health organization (WHO) designated it as the sixth public health emergency of international concern (PHEIC) and officially named it as COVID-19 (Jee, 2020). COVID-19 is caused by Severe Acute Respiratory Syndrome Corona Virus 2 (SARS-CoV-2), with common symptoms including fever, cough, and shortness of breath. In severe cases, pneumonia may occur and eventually lead to acute respiratory distress syndrome (ARDS), multiple organ failures, and even death (Huang et al., 2020). As of 16 January 2023, over 662 million confirmed cases of COVID-19 and over 6.7 million deaths have been reported worldwide (https://covid19.who.int/). Its negative impacts on the global economy and healthcare system are immeasurable and still ongoing. Healthcare systems in different countries are struggling to control the virus and prevent the spread of COVID-19 (Wang and Yang, 2021; Wang et al., 2022a; Jayk Bernal et al., 2022; Tomalka et al., 2022). Obviously, there is an urgent need to seek safe and effective drugs or adjunctive therapies against COVID-19 (Riva et al., 2020; Wang and Yang, 2020; 2022a; Yang and Wang, 2021; Wang et al., 2022b; Cully, 2022; Gao et al., 2022).

Various strategies, including the use of traditional medicine, have been considered to control the occurrence and development of COVID-19 (Ang et al., 2020). Traditional Tibetan medicine (TTM), one of the most famous traditional medicine systems in the world, has a long history of more than 3,800 years (Li, Q. et al., 2018). In long-term practice since ancient times, TTM has accumulated rich experience in the treatment of infectious diseases. Since the outbreak of COVID-19, Tibetan areas, including the Tibet Autonomous Region, Gansu and Qinghai, have issued multiple versions of the “Guidelines on the Diagnosis and Treatment of COVID-19,” which have played an important role in China’s fight against COVID-19 (Gyang et al., 2020). Some traditional Tibetan drugs (e.g., Liu Gan pills and Cui Tang granules) have been shown to relieve symptoms in patients with COVID-19 (Wang et al., 2021). Moreover, post-acute sequelae of SARS-CoV-2 infection (PASC) is a global health crisis that can have long-term adverse effects on the lungs and multiple extrapulmonary tissues and organs (Wang and Yang, 2022b). TTM herbs usually have the characteristics of multi-component and multi-target action, so they have protective effects on multiple organ damage of PASC. For example, Bawei Chenxiang pills can improve cardiovascular and cerebrovascular injury because of its functions of clearing heat and tranquilizing the mind (Commission, 1995; Zhu et al., 2011). In short, TTM has good advantages and promising prospects in the treatment of COVID-19 and PASC.

In this review, we briefly outline the history of Tibetan medicine fighting against the plague, summarize the TTM treatment strategies for COVID-19, and introduce 10 commonly used TTM drugs for preventing and treating COVID-19. Moreover, the related active components and mechanisms of action of these drugs were also analyzed and discussed. We hope this review will inform the public and professionals about TTM and its contribution to infectious diseases, and provide a reference for the development of new drugs against COVID-19.

## 2 The theoretical foundation of TTM for the treatment of COVID-19

### 2.1 The history of TTM in treating plague

TTM has a long history in preventing and treating plague. According to Tibetan medical history, in the 4th century AD, the Tubo king vBrong gnyan ldevu contracted an infectious disease similar to leprosy. To avoid infecting others, he voluntarily buried himself alive. This is the earliest recorded example of a plague-infected person being quarantined in the history of TTM (Suonan, 2009). The theoretical system of TTM for preventing and treating plague was firstly recorded in the classic book “*Si Bu Yi Dian* (*The Four Medical Tantras*)” written by Yutog Yontan Gonpo in the 8th century AD (Yuto, 1983; Wanme et al., 2021). It has described the etiology, pathogenesis, classification, and prevention of plague in detail. In addition, the “*Gan Lu Bao Ping*,” written in the 8th century AD, is regarded as a classic work and guide for TTM to treat plagues. Later, on the basis of the “*Si Bu Yi Dian*” and “*Gan Lu Bao Ping*,” Tibetan medicine experts from different generations compiled some classic books related to epidemic prevention, such as “*Mi Jue Xu Bu Yi*,” “*Mi Jue Hong Se Jin Han*, “*Shi Yi Liao Fa Ren Qing Sheng Ming Lian*,” and “*Mi Jue Bao Yuan*.” These monographs summarize the theory of TTM for epidemic prevention and record many classic prescriptions, such as “Jiuwei Heiyao Fangwen Powder” and “Shierwei Yishou Powder” (Suo and Chiren, 2020).

Since the outbreak of COVID-19, the health commissions in Tibetan ethnic areas have issued multiple editions of the “TTM Guidelines on the Diagnosis and Treatment of COVID-19” (Gyang et al., 2020). These guidelines describe the etiology, pathogenesis, and treatment principles of COVID-19 based on TTM theory, and recommend some traditional TTM drugs for the prevention and treatment of COVID-19 (Wang, X. et al., 2020). In June 2020, the Health Commission of the Tibet Autonomous Region convened a meeting to report the achievements of TTM in combating COVID-19. Some experts said that some TTM drugs used in Wuhan, such as Cui Tang granules, Liu Gan pills and Renqing Changjue capsules, have good therapeutic effects on COVID-19 (Zang, 2020). In August 2022, there was an outbreak of COVID-19 in the Tibet Autonomous Region of China. Subsequently, three makeshift hospitals were built in Lhasa, Shigatse, and Qamdo. The Lhasa makeshift hospital was equipped with a Tibetan medicine pharmacy, and TTM has become the main force in the treatment of COVID-19 (https://wjw.xizang. gov.cn/xwzx/wsjkdt/index_1.html). In short, the Tibetan people have continuously summed up their experience in the long history of fighting against the plague, formed a unique theoretical system of TTM and left many good prescriptions for the prevention and treatment of infectious diseases.

### 2.2 Understanding the pathogeny of COVID-19 in TTM theory

In the TTM theory, COVID-19 belongs to the category of “Nian Ran (གཉན་རིམས།)” disease (Li et al., 2022). “Nian Ran” is a general term for plagues with certain infectious ability. TTM theory believes that “Nian Ran” disease is closely related to evil-qi, season, behavior, and diet. The classic book “*Si Bu Yi Dian*” records: “Evil-qi and epidemic miasma spread all around. Seasonal irregularities, atrocities, anger, sadness, and improper diet and lifestyle may induce Nian Ran disease” (Yuto, 1983).Another TTM book “*Mi Jue Xu Bu Yi*” believes that “Nian Ran” disease is caused by the greedy attitude and improper behaviors of humankinds. They destroyed the ecological environment and awakened the “Babada” (པར་པ་ཏ།, similar to infectious microorganisms) in nature. Subsequently, “Babada” enters the human body through the nose and mouth, and competes with the inherent microorganisms in the body, and eventually causes “Nian Ran” disease (Wanme et al., 2021). Modern medicine believes that COVID-19 is mainly caused by the SARS-CoV-2 virus, which is similar to the “Babada” in TTM theory.

In addition, TTM’s understanding of disease mainly relies on the theory of “Three-Factor,” which refers to the “Loong (རླུང་།),” “Tripa (མཁྲིས་པ།),” and “Baekan (བད་ཀན།)” (Li, Q. et al., 2018). They are the three kinds of energy substances that maintain human life activities. In normal physiological state, they are interdependent and mutually restrained to achieve a balance. However, in a pathological state, the balance between the three in the body is disrupted, resulting in the disorders of the seven essences (ལུས་ཟུངས་བདུན།, including food essence, blood, muscle, fat, bone, marrow, and semen) and the three excrements (དྲི་མ་གསུམ།, including feces, urine and sweat), which further affects various organs of the body to develop disease (Yong and Ciren, 2015).

**FIGURE 1:**
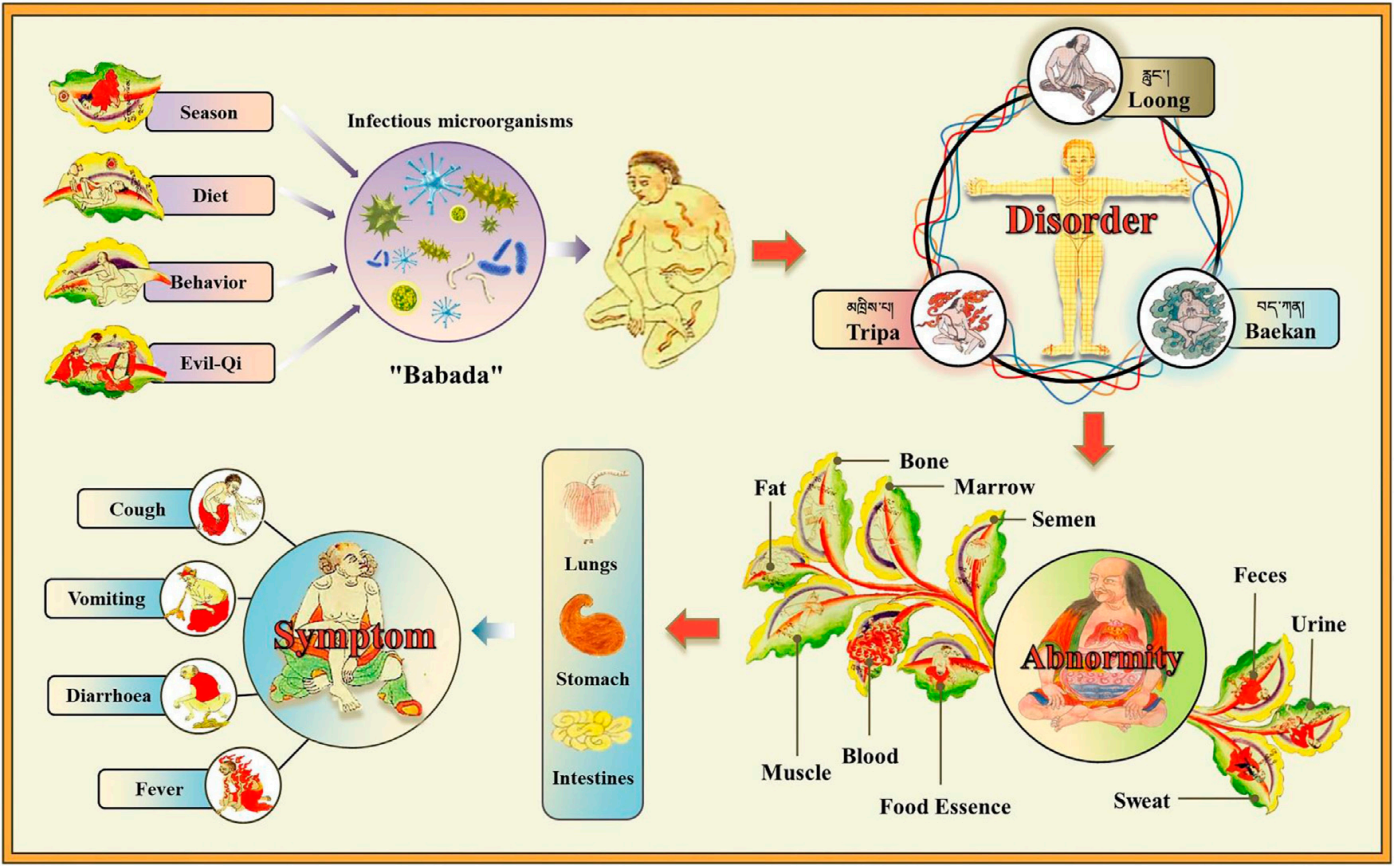
Understanding the pathogeny of COVID-19 in TTM theory. Due to the influence of diet, behavior, season, and evil-qi, “Babada” (similar to infectious microorganisms; here it refers to SARS-CoV-2) invades the human body. This causes the disorders of “Loong,” “Tripa,” and “Baekan,” which further leads to the abnormality of seven essences and three excrements. Subsequently, these changes gradually affect the lungs, stomach and intestines, causing symptoms such as cough, fever, diarrhea, and vomiting.

In conclusion, according to the TTM theory, COVID-19 is thought to be caused by “Babada” (including SARS-CoV-2) invasion due to improper diet, bad behavior, seasonal irregularities, and evil-qi accumulation. The invasion leads to the disorder of the “Three-Factor” in the body, which causes the abnormality of the seven essences and the three excrements. These changes gradually affect human organs including the lungs, stomach and intestines, resulting in symptoms such as cough, fever, diarrhea, and vomiting (Figure 1).

## 3 TTM treatment strategy for COVID-19

TTM divides COVID-19 into early, middle, late, and recovery periods, and proposes corresponding treatment plans based on the principles of plague treatment recorded in the classic book “*Gan Lu Bao Ping*.” These four periods are similar to the four stages of mild, moderate, severe, and rehabilitation stages divided by Western medicine according to the severity of COVID-19. The treatment strategies of TTM for the four stages are described in detail as follows.

(1) In the mild stage of COVID-19: SARS-CoV-2 replication occurs in the trachea and lungs, which may be incubated for 5–6 days (Wolfel et al., 2020). SARS-CoV-2 can invade epithelia cells by binding with Angiotensin Converting Enzymes 2 (ACE2) and transmembrane serine protease 2 (Dong et al., 2020). During this period, patients mainly present with symptoms such as fever and dry cough. This stage corresponds to the “early period” in TTM. The evil-qi of the virus has invaded the human body, but the “heat toxin” (a TTM term, similar to harmful substances) is not fully formed. The treatment strategy of TTM is to take ripening, diaphoresis, heat-clearing, and detoxifying drugs in time, aiming to accelerate ripening fever, weaken “heat toxin,” prevent deterioration, and alleviate symptoms.
(2) In the moderate stage of COVID-19: The viral infection continues to progress. This cause local inflammation and recruit immune cells from the blood to the lungs to eliminate extracellular viruses and destroy infected cells (Shi et al., 2020). Due to the excessive activation of immune cells in the lungs, a large number of cytokines are produced, and then an inflammatory storm is formed through a positive feedback loop (Demopoulos et al., 2020). This stage corresponds to the “middle period” in TTM. During this period, the “heat toxin” becomes severe, and patient presents a series of high fever symptoms. The treatment method of TTM is to take heat-clearing and detoxifying drugs, aiming to clear “heat toxin” and relieve symptoms.
(3) In the severe stage of COVID-19: During this period, severe endothelial injury, thrombus and microangiopathy occur, and patients rapidly develop into severe ARDS, acute lung injury, multiple organ dysfunction, and septic shock (Mihaila and Dragos Mihaila, 2020; Wan et al., 2020). This stage is similar to the “late period” in TTM. The treatment strategy of TTM is to take resuscitation, sedative and detoxification drugs, focusing on curing “Loong” disease, protecting organs, and reducing body disorders.
(4) In the rehabilitation stage of COVID-19: Most of the patients’ T-lymphocyte subsets, pulmonary function and chest CT have returned to normal, but the functional damage of immune, digestive, and cardiovascular systems is still not fully recovered (Chen, M. et al., 2021; Cui et al., 2021; Miao et al., 2022). This stage corresponds to the “recovery period” in TTM. It believes that although heat pathogens have been eliminated, it is also very necessary to use nourishing drugs to harmonize Qi and blood and viscera, aiming to supplement nutrition, promote the repair of damaged organs and tissues, enhance the body’s immunity, and prevent disease recurrence.

## 4 The commonly used TTM drugs for the prevention and treatment of COVID-19

**FIGURE 2:**
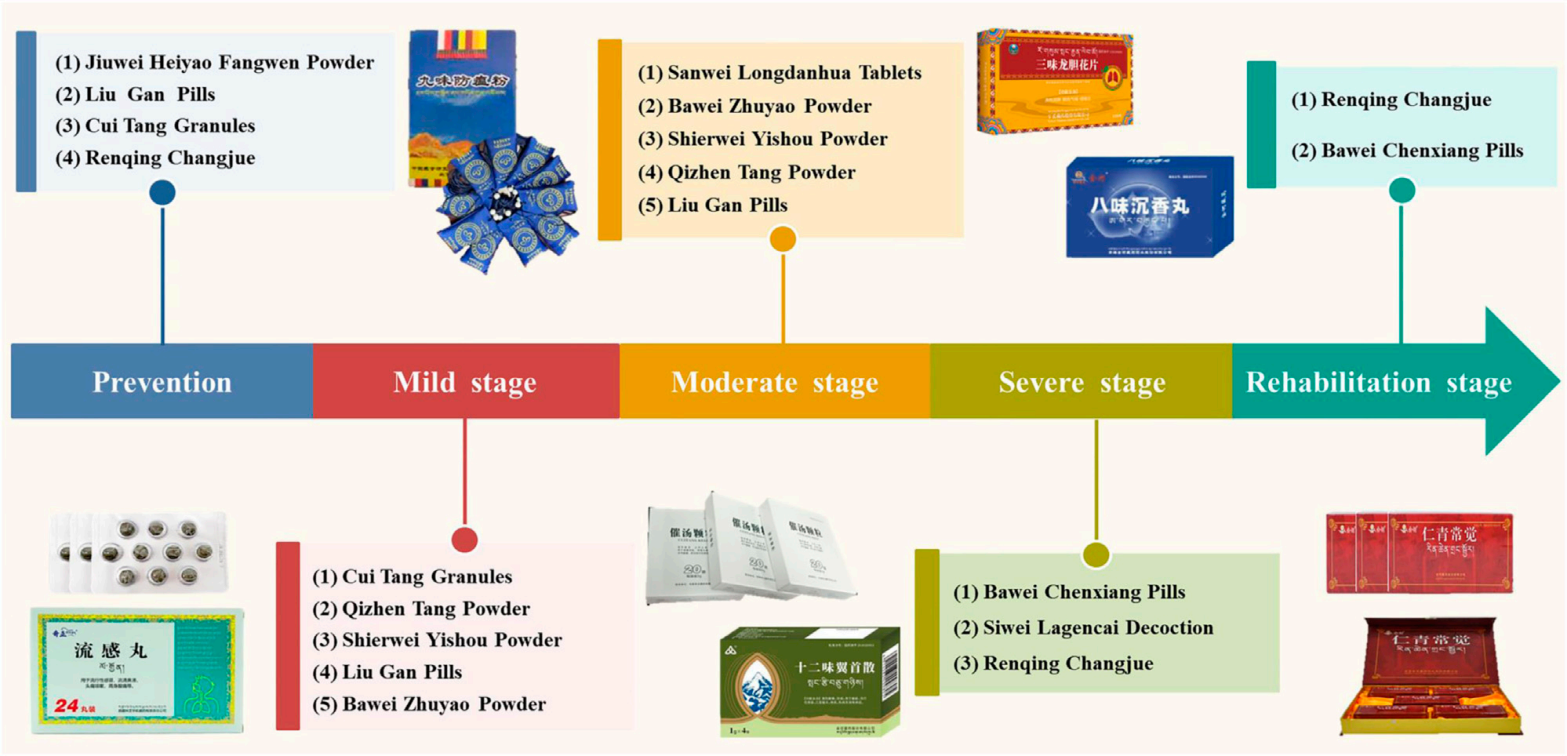
The most commonly used TTM drugs for the prevention and treatment of different stages of COVID-19.

In this review, we introduce the 10 most commonly used TTM drugs, including Jiuwei Heiyao Fangwen Powder, Liu Gan Pills, Cui Tang Granules, Qizhen Tang Powder, Bawei Zhuyao Powder, Sanwei Longdanhua Tablets, Bawei Chenxiang Pills, Siwei Lagencai Decoction, Shierwei Yishou Powder, and Renqing Changjue (Figure 2). The following will give a detailed introduction and discussion on the source, history of use, prescription composition, traditional functions and indications, and modern research progress of these Tibetan medicines. The information can provide important references for the development and utilization of these TTM drugs.

### 4.1 Jiuwei Heiyao Fangwen powder (ནགཔདགསར།, JHFP)

JHFP is a well-known plague-preventing drug in TTM medical system. It is derived from the classic Tibetan books “*Si Bu Yi Dian*” and “*Gan Lu Da Ping*.” The prescription of JHFP consists of nine Tibetan medicinal materials, including Anxixiang (The resin of *Anthostyrax tonkinensis* Pierre), Zangchangpu (The rhizome of *Acorus calamus* L*.*), Awei (The resin of *Ferula sinkiangensis* K. M. Shen or *Ferula fukanensis* K. M. Shen), Caowu (The roots of *Aconitum kusnezoffii* Rchb.), Xionghuang (Realgar), Dutousuan (The bulbs of *Allium sativum* L.), Niuhuang (The bile powder of *Bos Taurus domesticus* Gmelin), Honghua (The flowers of *Carthamus tinctorius* L.), and Shexiang (Moschus). In “Tibetan Medicine Standards,” JHFP is recorded to have the function of preventing various plagues (Health bureau of Tibet, 1979). It can be taken orally or made into sachets and medicine bags to wear. It was reported that JHFP has achieved good results in the prevention and control of Severe Acute Respiratory Syndrome (SARS) and Influenza Virus A (H1N1) (bKrashi and sMon, 2020). After the outbreak of COVID-19, JHFP was selected into the “TTM Guidelines on the Diagnosis and Treatment of COVID-19” in Tibet Autonomous Region, and played an important role in the prevention of COVID-19 in China. Wang et al. studied the antibacterial effect of JHFP by using air fumigation method, and found that it has antibacterial effects against *Pseudomonas aeruginosa*, *Salmonella paratyphoid*, and *Staphylococcus aureus* (Wang et al., 2013).

### 4.2 Liu Gan Pills (ལགནརལབ།, LGP)

LGP is a commonly used Tibetan medicine for preventing and treating epidemics. In the past 3 years, LGP has been widely used in the prevention and treatment of COVID-19 in Tibetan areas of China, including Tibet Autonomous Region, Qinghai Province, and Gansu Province. LGP is consisted of 21 herbs (Commission, 1995), including Hezi (The fruits of *Terminalia chebula* Retz.), Yadahuang (The roots and rhizomes of *Rheum spiciforme* Royle or other plants of the same genus), Muxiang (The roots of *Aucklandia lappa* Decne.), Zhangyacai (The whole grass of *Swertia bimaculata* (Siebold and Zucc.) Hook. f. and Thomson ex C. B. Clarke), Zangmuxiang (The roots of *Inula racemosa* Hook.f.), Chuitouju (The flowers of *Cremanthodium lineare* Maxim.), Dingxiang (The flower buds of *Eugenia caryophyllata* Thunb.), Lianxingjidou (The herbs of *Oxytropis falcata* Bunge), Suantengguo (The fruits of *Embelia oblongifolia* Hemsl.), Jiaohuixiang (The herbs of *Hypecoum erectum* L*.*), Awei (The resin of *F. sinkiangensis* K. M. Shen or *F. fukanensis* K. M. Shen), Bangga (The herbs of *Aconitum naviculare* (Brühl) Stapf or *Aconitum tanguticum* (Maxim.) Stapf), Daji Gao (The extract of root tubers of *Euphorbia micractina* Boiss.), Caowu (The roots of *A. kusnezoffii* Rchb.), Anxixiang (The resin of *A. tonkinensis* Pierre), Zangchangpu (The rhizome of *A. calamus* L*.*), Longgu (*Os Draconis*), Shexiang (Moschus), Kuangjinteng (The stems of *Tinospora cordifolia* (Willd.) Miers or *Tinospora sinensis* (Lour.) Merr.), Niuhuang (The bile powder of *Bos Taurus* domesticus Gmelin), and Doukou (The fruits of *Amomum kravanh* Pierre ex Gagnep. or *Amomum compactum* Sol. ex Maton). In “Drug Standards of Tibetan Medicine,” LGP is recorded with heat-clearing and detoxifying effects, and is commonly used to treat influenza, headache, cough, body aches, and fever (Commission, 1995). Wang et al. studied the clinical efficacy of LGP on COVID-19, and the results showed that LGP could relieve the symptoms of fever, nasal obstruction, runny nose, chest tightness, and diarrhea in patients with COVID-19 (5 cases) (Wang et al., 2021). However, there are currently no reports on the active ingredients and therapeutic mechanisms of LGP against COVID-19, and further research is needed.

### 4.3 Cui Tang Granules (འཕལཐང།, CTG)

CTG is made from the classic Tibetan medicine Cui Tang Pills (CTP) by changing the drug form. CTP has a history of more than 600 years in clinical application, and its prescription comes from the monograph “*Yi Xu Qian Wan She Li*” written by the famous Tibetan doctor Sukar Nangni Dorje. CTP and CTG are commonly used drugs in the TTM medical system for the prevention and treatment of influenza and cold. In 2020, in the fight against COVID-19, CTG passed the emergency filing of Gansu Food and Drug Administration, and then this drug was quickly used in the prevention and control of the epidemic. CTP is now recorded in the 2020 edition of the *Chinese Pharmacopoeia* (Commission, 2020). The prescription is composed of Zangmuxiang Gao (The extract of roots of *I. racemosa* Hook.f.), Zangmuxiang (The roots of *I. racemosa* Hook.f.), Xuangouzi Jing (The stems of *Rubus saxatilis* L. or *Rubus biflorus* Buch.-Ham. ex Sm.), Kuanjinteng (The stems of *T. sinensis* (Lour.) Merr.), GanJiang (The rhizomes of *Zingiber officinale* Roscoe), Hezi Rou (The pulp of *T. chebula* Retz.), Yuganzi (The fruits of *Phyllanthus emblica* L.), Maohezi (The fruits of *Terminalia bellirica* (Gaertn.) Roxb.), and Pangxiejia (The root tubers of *Phlomis younghusbandii* Mukerjee). It has the functions of clearing heat, relieving cough and alleviating pain, and can be used to prevent influenza and treat cough, headache, and joint pain caused by cold (Commission, 2020).

Wang et al. evaluated the clinical efficacy of CTG in the treatment of COVID-19 (Wang et al., 2021). The results showed that CTG could improve the symptoms of fever, cough, fatigue, sore throat, and diarrhea in patients with COVID-19 (8 cases). In addition, Ma et al. studied the clinical efficacy of CTG on influenza, and found that it has a good therapeutic effect with a total effective rate of 92.86% (Ma et al., 2018). Another study found that CTG could improve symptoms such as headache, runny nose, and joint pain in patients with acute upper respiratory tract infection, with a total effective rate of 94.44% (Basang et al., 2017). Recently, Chen et al. studied the mechanism of CTG in the treatment of COVID-19 through network pharmacology and molecular docking methods (Chen, L.Y. et al., 2021). The results showed that CTG could bind to the 3CL hydrolase of SARS-CoV-2 and ACE2 through its active components, and act on viral infection, hypoxia-inducible factor-1, Tumor Necrosis Factor (TNF), and Vascular Endothelial Growth Factor (VEGF) signaling pathways, thereby improving COVID-19.

### 4.4 Qizhen Tang powder (འཕལཐང།, QTP)

QTP is derived from the classic prescriptions Siwei Zangmuxiang Tang Powder (མནབཞཐང།, SZTP) and San Guo Tang Powder (འབ;སབགསམཐང།, SGTP). Currently, QTP is recorded in the “Drug Standards of Tibetan medicine.” It has the functions of dispelling cold and relieving exterior, and is often used to treat anemofrigid cold, initial onset of fever, malignant fever, and joint pain (Commission, 1995). QTP consists of seven Tibetan herbs, including Zangmuxiang (The roots of *I. racemosa* Hook.f.), Xuangoumu (The stems of *R. saxatilis* L. or *R. biflorus* Buch.-Ham. ex Sm.), Kuanjinteng (The stems of *T. sinensis* (Lour.) Merr.), Ganjiang (The rhizomes of *Z. officinale* Roscoe), Hezi (The fruits of *T. chebula* Retz.), Maohezi (The fruits of *T. bellirica* (Gaertn.) Roxb.), and Yuganzi (The fruits of *P. emblica* L.). Among them, four herbs (Zangmuxiang, Xuangoumu, Kuanjinteng, and Ganjiang) make up SZTP. It has the functions of relieving exterior and sweating, and can be used to treat chills, headaches, joint pains, and fever in the early stage of plague or influenza (Commission, 1995). Moreover, the other three herbs (Hezi, Maohezi, and Yuganzi) make up SGTP. It can clear heat and harmonize qi-blood, and is often used to treat plague fever and overwork (Health bureau of Tibet, 1979). So far, there are few studies on the clinical efficacy and pharmacological activity of QTP in the treatment of infectious diseases. Only one study used QTP to treat 68 elderly patients with influenza, and the results showed that the total effective rate was 89% (Mo, 2019).

### 4.5 Bawei Zhuyao powder (གཙབབརདཔ།, BZP)

BZP is one of the recommended drugs for the treatment of COVID-19 in the “TTM Guidelines on the Diagnosis and Treatment of COVID-19” in Tibet Autonomous Region of China. It is currently recorded in the “Drug Standards of Tibetan medicine.” BZP is slightly fragrant and bitter in flavor, and has the effects of clearing heat and detoxifying. It is frequently used in the TTM medical system for the treatment of febrile diseases, such as distemper, lung heat, blood heat, and liver heat ^[30]^. BZP consists of Niuhuang (The bile powder of *Bos T. domesticus* Gmelin), Tanxiang (The heartwood of *Santalum album* L.), Tianzhuhuang (The exudates of *Bambusa textilis* McClure or *Schizostachyum chinense* Rendle), Honghua (The flowers of *C. tinctorius* L.), Zhangyacai (The herbs of *Swertia purpurascens* (D.Don) C. B. Clarke or other plants of the same genus), Baxiaga (The branchs and leaves of *Adhatoda vasica* Nees), Tuercao (The herbs of *Lagotis brevituba* Maxim*.*), and Bangga (The herbs of *A. naviculare* (Brühl) Stapf or *A. tanguticum* (Maxim.) Stapf). Among them, Niuhuang can expel the plague and detoxify, Tanxiang can clear heat and moisten the lungs, Honghua can promote blood circulation and dredge the meridians, and the other five herbs have the effects of clearing heat and detoxifying (Commission, 1995). To sum up, from the perspective of the functions and indications of BZP and its constituent herbs, it has a promising curative effect on COVID-19 or other infectious diseases. However, to date, no studies have been performed to evaluate the clinical efficacy and pharmacological activity of BZP.

### 4.6 Sanwei Longdanhua tablets (སངརནགསམཔ།, SLT)

SLT is a commonly used Tibetan medicine, and is recommended for the treatment of COVID-19 in the “Technical Guidelines for the Prevention and Control of COVID-19” in Sichuan Province, China. Its prescription consists of Baihualongdan (The flowers of *Gentiana szechenyii* Kanitz), Gancao (The roots and rhizomes *Glycyrrhiza uralensis* Fisch. or *Glycyrrhiza inflata* Batalin or *Glycyrrhiza glabra* L.), and Fengmi (Honey). SLT has the functions of clearing heat, moistening the lungs, and soothing the throat, and can treat lung heat, pharyngitis and other “Nian Ran” related diseases (Zhao et al., 2020). Baihualongdan is the monarch drug in SLT prescription. It has the effects of purging fire, clearing damp heat and relieving cough, and is often used to treat lung heat or other respiratory diseases (Zong et al., 2015). Moreover, Gancao has the functions of clearing heat, detoxifying, dispelling phlegm, and relieving cough, while Fengmi can nourish the spleen for nourishing qi, detoxify and relieve pain (Commission, 2020).

One study found that SLT had a protective effect on rats with acute pneumonia by inhibiting Nuclear Factor kappa-B (NF-κB) signaling pathway (Li, Y.Q. et al., 2018). It could significantly alleviate lung tissue damage, reduce the serum levels of Tumor Necrosis Factor-alpha (TNF-α), Interleukin-1 beta (IL-1β) and Interferon-γ (IFN-γ), and inhibit the phosphorylation of NF-κB p65. In addition, Zhao et al. studied the potential mechanisms and active components of SLT in the treatment of COVID-19 by using network pharmacology and molecular docking methods (Zhao et al., 2020). The results showed that quercetin and kaempferol present in SLT could bind to the RNA-dependent RNA polymerase (RdRp), Main protease (Mpro) and ACE2, and regulate Mitogen-Activated Protein Kinase (MAPK), arachidonic acid metabolism and calcium signaling pathways, thereby improving COVID-19.

### 4.7 Bawei Chenxiang pills (ཨགརབརདཔ།, BCP)

BCP was first recorded in the classic book “*Si Bu Yi Dian*,” and has been used clinically for more than 1,300 years. It is now recorded in the “Drug Standards of Tibetan medicine.” BCP consists of eight medicinal materials, including Chenxiang (The resinous wood of *Aquilaria sinensis* (Lour.) Spreng.), Roudoukou (The seed kernels of *Myristica fragrans* Houtt.), Guangzao (The fruits of *Choerospondias axillaris* (Roxb.) B. L. Burtt and A. W. Hill), Hezi (The fruits of *T. chebula* Retz.), Ruxiang (The resin of *Boswellia carteri* Birdw. or *Boswellia bhaw-dajiana* Birdw.), Muxiang (The roots of *A. lappa* Decne.), Mumianhua (The flowers of *Gossampinus malabarica* Merr.), and Shihuihua (Calcsinter). It possesses the effects of clearing heart heat, tranquilizing the mind and inducing resuscitation, and is often applied to treat fever, delirium, precordial pain, and cardiac trauma (Commission, 1995). Chenxiang is the monarch drug in BCP prescription. It has been proven to have anti-myocardial ischemia, antitussive, antiasthmatic, anti-inflammatory, and sedative activities (Li et al., 2019), and is widely used in the treatment of cardiovascular and cerebrovascular, respiratory, and urinary system diseases.

It has been reported that patients with COVID-19 are prone to acute myocardial injury, arrhythmia and acute stroke, among which myocardial injury is a risk factor for in-hospital death in critically ill patients with COVID-19 (Larson et al., 2020; Wang, L.Y. et al., 2020). BCP is often used in Tibetan medicine to treat various cardiovascular and cerebrovascular diseases. It has been proved to have a protective effect on myocardial ischemia injury, and can also improve cardiac and cerebral ischemia-reperfusion injury (Zhu et al., 2011; Zhu et al., 2019). Therefore, taking BCP may be beneficial to improve the cardiovascular and cerebrovascular damage caused by COVID-19.

### 4.8 Siwei Lagencai decoction (སལིཐང།, SLD)

SLD, also known as “Suoluoxi Decoction,,” its formula and efficacy are recorded in detail in the “*Si Bu Yi Dian*.” It is consisted of four herbs, including Gaoshanlagencai (The roots of *Pegaeophyton scapiflorum* (Hook.f. and Thomson) C. Marquand and Airy Shaw), Zicaorong (The resin produced by *laccifer lacca* Keer.), Ligadu (The roots and rhizomes of *Bergenia purpurascens* (Hook.f. and Thomson) Engl.), and Gancao (The roots and rhizomes of *G. uralensis* Fisch. or *G. inflata* Batalin or *G. glabra* L.). SLD has the functions of clearing lung heat, eliminating phlegm and relieving cough, and is often applied to treat lung heat, cough, excessive phlegm, and fever (Commission, 1995). In the SLD prescription, Gaoshanlagencai has the effects of relieving lung heat, preventing plague and hemostasis, while Ligadu can prevent plague, relieve lung heat, and reduce swelling (Wencheng and Dong, 2018). At present, there are few studies on the pharmacological activity and clinical efficacy of SLD. Suonan et al. found that SLD could significantly improve cough and expectoration symptoms in patients with colds, pneumonia or tuberculosis after 10 days of administration, with a total effective rate of 93.3% (Suonan and Dajie, 2015).

### 4.9 Shierwei Yishou powder (སངརབཅགཉས།, SYP)

SYP is a commonly used Tibetan medicine, and is recommended for the treatment of COVID-19 in the “TTM Guidelines on the Diagnosis and Treatment of COVID-19.” SYP is now recorded in the 2020 edition of the *Chinese Pharmacopoeia*. It has the functions of clearing heat, detoxifying, and preventing epidemics, and is often used to treat plague, influenza, dysentery, and fever (Commission, 2020). SYP prescription consists of 12 Tibetan medicines, including Yishoucao (The herbs of *Pterocephalus hookeri* (C. B. Clarke) E. Pritz.), Bangga (The herbs of *A. naviculare* (Brühl) Stapf or *A. tanguticum* (Maxim.) Stapf), Jieliejiaohuixiang (The herbs of *Hypecoum leptocarpum* Hook. f. and Thomson), Tianzhuhuang (The exudates of *B. textilis* McClure or *S. chinense* Rendle), Honghua (The flowers of *C. tinctorius* L.), Tanxiang (The heartwood of *Santalum album* L.), Anxixiang (The resin of *A. tonkinensis* Pierre), Edaxia (The herbs of *Oxytropis falcata* Bunge or *Oxytropis microphylla* (Pall.) DC.), Wulingzhi Gao (The extract of faeces of *Trogopterus xanthipes* Milne-Edwards), Tiebangchuiye (The leaves of *Aconitum flavum* Hand.-Mazz. or *Aconitum pendulum* N.Busch), Niuhuang (The bile powder of *Bos T. domesticus* Gmelin), and Shexiang (Moschus). Among them, Yishoucao is the monarch drug of SYP. It possesses the functions of detoxifying, eliminating plague, clearing heat, dispelling wind, and relieving arthralgia (Commission, 2020). Moreover, Yishoucao has been proved to have anti-inflammatory, analgesic, anti-rheumatoid arthritis, and neuroprotective activities (Gan et al., 2021).

Recently, Zhang et al. explored the potential mechanisms of SYP in the treatment of COVID-19 through network pharmacology technology (Zhang et al., 2021). The results showed that SYP could act on some targets such as TNF, IL-6, glyceraldehyde-3-phosphate dehydrogenase, MAPK3, and epidermal growth factor receptor, and regulate immune response, inflammation, viral infection, and endothelial cell function signaling pathways, thereby improving COVID-19.

### 4.10 Ren Qing Chang Jue (རནཆནགངསར།, RQCJ)

RQCJ is a precious Tibetan medicine. It was first recorded in the “*Si Bu Yi Dian*,” and has been used clinically for more than a thousand years. At present, RQCJ is recorded in the 2020 edition of the *Chinese Pharmacopoeia*. The prescription of RQCJ consists of many Tibetan medicines, including Zhenzhu (Pearl), Zhusha (Cinnabar), Tanxiang (The heartwood of *Santalum album* L.), Chenxiang (The resinous wood of *Aquilaria sinensis* (Lour.) Spreng.), Niuhuang (The bile powder of *Bos T. domesticus* Gmelin), Shexiang (Moschus), Hezi (The fruit of *T. chebula* Retz.), Xihonghua (The stigma of *Crocus sativus* L.), etc. RQCJ is slightly fragrant and sweet in flavor, and has the functions of clearing heat, detoxifying, and nourishing the body (Commission, 2020).

It was reported that RQCJ had obvious analgesic, anti-fatigue and immune-enhancing effects (Sun et al., 2014). Specifically, it could significantly increase the pain threshold of mice, prolong the weight-bearing swimming time of mice, and enhance the phagocytic function of phagocytes in mice with spleen deficiency. In addition, Gao et al. found that RQCJ had a protective effect on lipopolysaccharide (LPS)-induced injury of alveolar type II epithelial cells in rats, and its mechanisms might be related to reducing the release of inflammatory factors (IL-6 and TNF-α) and resisting apoptosis (Gao et al., 2021).

## 5 Analysis of the therapeutic mechanism of TTM drugs for COVID-19

**FIGURE 3:**
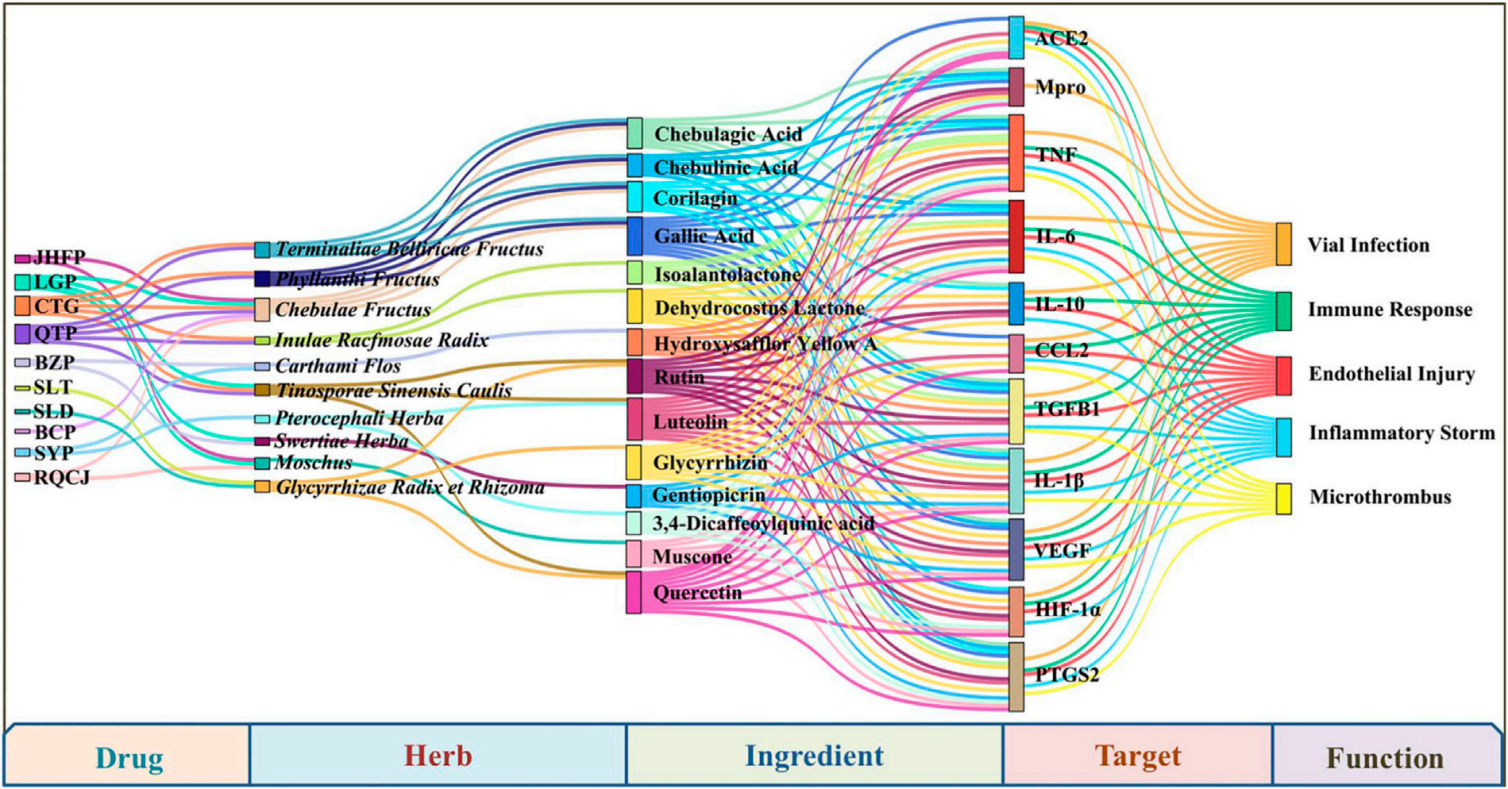
The drug-herb-ingredient-target-function network of frequently used herbs in recommended prescriptions and their main ingredients, as well as their key targets and functions for COVID-19.

Based on the prescriptions of the above 10 drugs, we found that Hezi, Maohezi, Yuganzi, Gancao, Zangmuxiang, Honghua, Yishoucao, Kuanjinteng, Zhangyacai, and Shexiang are the most commonly used TTM herbs for the prevention and treatment of COVID-19. In order to explore the therapeutic mechanism of TTM for COVID-19, this review established a drug-herb-ingredient-target-function network using Omicshare software, including 10 TTM drugs, 10 representative herbs, 14 chemical constituents, 11 key targets, and five involved functions (Figure 3). Among them, 14 chemical constituents, such as gallic acid, chebulagic acid, rutin, luteolin, and glycyrrhizicn, may play an important role in the treatment of COVID-19 with TTM. The active ingredients in TTM that have been shown to exhibit activity against SARS-Cov-2 *in vitro* are shown in Table 1.

**TABLE 1:** Summary of the active ingredients from TTM that have been shown to exert activity against SARS-Cov-2 *in vitro*.

No.	Compound	TTM	EC_50_ or IC_50_	Reference
1	Chebulagic acid	Liu Gan Pills, Cui Tang Granules, Qizhen Tang Powder, Bawei Chenxiang Pills, Ren Qing Chang Jue	9.76 μM	Du et al. (2021)
2	Corilagin	Liu Gan Pills, Cui Tang Granules, Qizhen Tang Powder, Bawei Chenxiang Pills, Ren Qing Chang Jue	24.9 μM	Yang et al. (2021)
3	Gallic acid	Liu Gan Pills, Cui Tang Granules, Qizhen Tang Powder, Bawei Chenxiang Pills, Ren Qing Chang Jue	108 μg/mL	El Gizawy et al. (2021)
4	Rutin	Liu Gan Pills, Sanwei Longdanhua Tablets, Siwei Lagencai Decoction	31 μg/mL	El Gizawy et al. (2021)
5	Luteolin	Liu Gan Pills, Shierwei Yishou Powder	8.817 μmol/L	Xiao et al. (2022)
6	Glycyrrhizin	Sanwei Longdanhua Tablets, Siwei Lagencai Decoction	0.53 μM	Sand et al. (2020)
7	Quercetin	Liu Gan Pills, Sanwei Longdanhua Tablets, Siwei Lagencai Decoction	52.98 μmol/L	Xiao et al. (2022)

Gallic acid is a common phenolic acid compound present in Hezi, Maohezi, and Yuganzi. It has been shown to have anti-inflammatory, antioxidant and antiviral effects (Bai et al., 2021; Junior et al., 2021). Gallic acid was found to inhibit the release of some inflammatory factors (TNF-α, IL-1β, and IL-6) and cytokines (CCL2, PTGS2, and NO) by regulating NF-κB and MAPK signaling pathways (Kahkeshani et al., 2019). Furthermore, gallic acid could ameliorate chronic obstructive pulmonary disease-related exacerbations in mice by reducing proinflammatory cytokine levels and normalizing redox imbalance in the lungs (Singla et al., 2021). Molecular docking analysis indicated that gallic acid might inhibit SARS-CoV-2 cell entry through the ACE2 receptor and inhibit the proteolytic process (Arokiyaraj et al., 2020). Gizawy et al. found that gallic acid had remarkable anti-SARS-Cov-2 activity *in vitro* with an IC50 value of 108 μg/mL (El Gizawy et al., 2021).

Chebulagic acid, a hydrolysable polyphenolic compound, has been recognized as a broad-spectrum antiviral compound (Lin et al., 2011; Lin et al., 2013; Zhang et al., 2018). It was reported that chebulagic acid could downregulate the protein expression of inducible Nitric Oxide Synthase (iNOS), Cyclooxygenase-2 (COX-2) and Prostaglandin E2 (PGE2), thereby inhibiting the inflammatory response caused by syncytial virus (Lin et al., 2013). Moreover, chebulagic acid inhibited SARS-CoV-2 replication *in vitro* by targeting viral 3-Chymotrypsin-Like Cysteine protease (3CLpro) in reversible non-competitive manner (Du et al., 2021).

Glycyrrhizicn is the main active ingredient in Gancao. Luo et al. believe that glycyrrhizicn may be an effective therapeutic agent for COVID-19 due to its diverse pharmacological activities, including binding to ACE2, down-regulating proinflammatory cytokines, inhibiting the accumulation of intracellular reactive oxygen species, and inhibiting the hyperproduction of airway exudates (Luo et al., 2020). Two studies reported that glycyrrhizin could block viral replication by inhibiting Mpro, the main protease of SARS-CoV-2 (Tolah et al., 2021; van de Sand et al., 2021). In addition, an *in vitro* study demonstrated that glycyrrhizin could interfere with virus entry by directly interacting with ACE2 and spike protein (Diomede et al., 2021).

Luteolin is a flavonoid compound present in both Yishoucao and Kuanjinteng. It has been shown to have significant antiviral, antioxidant, neuroprotective, cardioprotective, and anti-inflammatory effects (Taheri et al., 2021). Through system pharmacology and bioinformatics analysis, Xie et al. reported that luteolin might exert effects on virus defense, regulation of inflammation and immune responses, and reduction of oxidative stress (Xie et al., 2021). Furthermore, several *in vitro* studies found that luteolin could specifically bind to the surface spike protein of SARS-Cov-2 to prevent virus entry into cells, and could also inhibit cytokine storm caused by IL-1β and histamine (Yi et al., 2004; Jo et al., 2020).

Rutin, a flavonoid present in Gancao and Kuanjinteng, has been reported to have antiviral and protective effects on blood vessels and lung tissue (Ganeshpurkar and Saluja, 2017). Ortolani et al. found that rutin were efficient in protecting the lungs of patients with early adult respiratory distress syndrome (Ortolani et al., 2000). In a molecular docking study, rutin showed significant binding to the Mpro, RdRp, Papain-Like protease (PLpro), and S-proteins of SARS-CoV-2 (Rahman et al., 2021). Recently, rutin was found to have remarkable anti-SARS-CoV-2 activity with an IC_50_ value of 31 μg/mL, and significant anti-inflammatory effects by reducing the levels of TNF-α, IL-1β, IL-2, and granulocyte colony-stimulating factor (El Gizawy et al., 2021).

In conclusion, the potential mechanisms of TTM for the treatment of COVID-19 include, but are not limited to, targeting ACE2 or 3Clpro to inhibit the invasion and replication of SARS-Cov-2, thereby preventing virus infection. In addition, TTM can regulate immune function and inflammatory response by reducing the levels of some cytokines such as IL-6, IL-1β, TNF-α, CCL2, and PTGS2, thereby improving excessive immunity, cytokine storm, endothelial damage and microthrombosis caused by COVID-19. According to current research reports, the active ingredients that produce these biological activities may be 14 compounds, including chebulagic acid, rutin, gallic acid, luteolin, and glycyrrhizicn (Figure 3). However, special attention should also be paid to other characteristic compounds present in these 10 Tibetan herbs, such as hydroxysafflor yellow A, swertiamarin, gentiopicrin, tinosineside A, cantleyoside, and sylvestroside I. These compounds may have the potential to improve COVID-19 because of their high concentration levels in the corresponding Tibetan herbs. More *in vitro* and *in vivo* studies are needed to explore the therapeutic potential of these compounds against COVID-19.

## 6 Conclusion and prospects

After the outbreak of COVID-19, scientists worldwide are searching for safe and effective treatments and drugs to slow the spread of the disease and reduce the morbidity and mortality of COVID-19. Although the development and use of anti-COVID-19 vaccines have brought hope for disease control, the threat of virus mutation and new epidemics still exists, and it is urgent and reasonable to continue to find effective intervention strategies and therapeutic drugs (Ren et al., 2020). TTM is one of the oldest known traditional medical systems in the world, with a very complete and unique theoretical system. The “Nian Ran” theory in the TTM system provides unique insights into the prevention and treatment of various plagues. Therefore, TTM has played an important role in the fight against epidemics in recent decades. Since ancient times, TTM has developed many classic drugs to treat infectious diseases in clinical practice, such as JHFP, LGP, CTG, QTP, BZP, and SLD. Among them, the efficacy of LGP and CTG in the treatment of COVID-19 has been confirmed by modern clinical studies.

Vaccination is an effective method to prevent SARS-CoV-2 infection. However, the emergence of virus variants, including Alpha, Beta, Gamma, Delta, and Omicron variants, poses a challenge to the efficacy of current vaccines. In addition, the post-acute sequelae of COVID-19 also have a long-term impact on human health. Therefore, in addition to treating COVID-19, there is an urgent need to find drugs that are also effective against the SARS-CoV-2 variant and the sequelae of COVID-19. Through therapeutic mechanism analysis, we found that TTM drugs have the characteristics of multi-component and multi-target action, and can effectively treat viral infection, excessive immunity, cytokine storm, endothelial injury, and microthrombosis caused by COVID-19 (Figure 3). Notably, some phytochemicals in TTM drugs, such as rutin and quercetin, also have good inhibitory effects on the Gamma and Delta variants of SARS-CoV-2 (Ansari et al., 2022). Moreover, some TTM drugs have good potential to improve the sequelae of COVID-19. For example, BCP can improve cardiovascular and cerebrovascular injury, and RQCJ can fight fatigue and improve immunity (Zhu et al., 2011; Sun et al., 2014). In summary, TTM may play a role in multiple stages of COVID-19 and has promising prospects for treating COVID-19 and its sequelae. However, the modern research on these Tibetan medicines is in its infancy, and there are still some problems to be solved. For example, most of the current research is just *in vitro* tests or computer virtual screening. More *in vivo* experiments are needed to reveal their active ingredients and molecular mechanisms against COVID-19 with the help of pharmacology and/or metabonomics methods. Moreover, there may be some valuable compounds in the most commonly used TTM herbs, especially those in high concentrations. Therefore, more studies are needed to explore the therapeutic value of several characteristic compounds for COVID-19, including hydroxysafflor yellow A, swertiamarin, gentiopicrin, tinosineside A, cantleyoside, and sylvestroside I.

This review aims to provide health workers with information to better understand the history and current status of TTM in treating infectious diseases, especially COVID-19. Tibetan medicine is a treasure of China’s and even the world’s medical system, and can provide valuable experience and more options for the prevention and treatment of COVID-19 in the future. The 10 commonly used drugs might be a precious gift from the old Tibetan medicine to the world, and might have potential as drug candidates for the treatment of COVID-19. These treasures urgently need to be excavated using modern scientific methods.

## Glossary

3CLpro: 3-Chymotrypsin-Like Cysteine proteas
ACE2: Angiotensin Converting Enzymes 2
ARDS: Acute Respiratory Distress Syndrome
BCP: Bawei Chenxiang Pills
BZP: Bawei Zhuyao Powder
COVID-19: The Coronavirus Diseases 2019
COX-2: Cyclooxygenase-2
CTP: Cui Tang Pills
H1N1: Influenza Virus A
IFN-γ: Interferon-γ
IL-1β: Interleukin-1 beta
iNOS: inducible Nitric Oxide Synthase
JHFP: Jiuwei Heiyao Fangwen Powder
LGP: Liu Gan Pills
MAPK: Mitogen-Activated Protein Kinase
Mpro: Main protease
NF-κB: Nuclear Factor kappa-B
PGE2: Prostaglandin E2
PHEIC
PLpro: Papain-Like protease
Public Health Emergency of International Concern
QTP: Qizhen Tang Powder
RdRp: RNA-dependent RNA polymerase
RQCJ: Ren Qing Chang Jue
SARS: Severe Acute Respiratory Syndrome
SARS-CoV-2: Severe Acute Respiratory Syndrome Corona Virus 2
SGTP: San Guo Tang Powder
SLD: Siwei Lagencai Decoction
SLT: Sanwei Longdanhua Tablets
SYP: Shierwei Yishou Powder
SZTP: Siwei Zangmuxiang Tang Powder
TNF: Tumor Necrosis Factor
TNF-α: Tumor Necrosis Factor-alpha
TTM: Traditional Tibetan medicine
VEGF: Vascular Endothelial Growth Factor
WHO: The World Health Organization.
